# Rapid identification of enteric bacteria from whole genome sequences using average nucleotide identity metrics

**DOI:** 10.3389/fmicb.2023.1225207

**Published:** 2023-12-14

**Authors:** Rebecca L. Lindsey, Lori M. Gladney, Andrew D. Huang, Taylor Griswold, Lee S. Katz, Blake A. Dinsmore, Monica S. Im, Zuzana Kucerova, Peyton A. Smith, Charlotte Lane, Heather A. Carleton

**Affiliations:** Centers for Disease Control and Prevention, Division of Foodborne, Waterborne and Environmental Diseases, National Center for Emerging and Zoonotic Infectious Diseases, Atlanta, GA, United States

**Keywords:** average nucleotide identity, ANI, species identification, enteric bacteria, WGS

## Abstract

Identification of enteric bacteria species by whole genome sequence (WGS) analysis requires a rapid and an easily standardized approach. We leveraged the principles of average nucleotide identity using MUMmer (ANIm) software, which calculates the percent bases aligned between two bacterial genomes and their corresponding ANI values, to set threshold values for determining species consistent with the conventional identification methods of known species. The performance of species identification was evaluated using two datasets: the Reference Genome Dataset v2 (RGDv2), consisting of 43 enteric genome assemblies representing 32 species, and the Test Genome Dataset (TGDv1), comprising 454 genome assemblies which is designed to represent all species needed to query for identification, as well as rare and closely related species. The RGDv2 contains six *Campylobacter* spp., three *Escherichia/Shigella* spp., one *Grimontia hollisae*, six *Listeria* spp., one *Photobacterium damselae*, two *Salmonella* spp., and thirteen *Vibrio* spp., while the TGDv1 contains 454 enteric bacterial genomes representing 42 different species. The analysis showed that, when a standard minimum of 70% genome bases alignment existed, the ANI threshold values determined for these species were ≥95 for *Escherichia*/*Shigella* and *Vibrio* species, ≥93% for *Salmonella* species, and ≥92% for *Campylobacter* and *Listeria* species. Using these metrics, the RGDv2 accurately classified all validation strains in TGDv1 at the species level, which is consistent with the classification based on previous gold standard methods.

## Introduction

Conventional bacterial species identification methods, such as phenotypic testing and gene-sequencing analysis, have been employed within the scientific community for years. However, with the increased use of next generation sequencing, new methods are available to analyze the entire DNA of the organisms. This allows for the simultaneous capture of a wide range of information, including whole genes, core genes, and ribosomal genes for species identification and strain typing, characterization of genes for serotype, virulence, antimicrobial resistance, kmer-typing, and much more (Jolley et al., 2012; Besser et al., 2018; Gerner-Smidt et al., 2019a, Gerner-Smidt et al., 2019b; Stevens et al., 2022). More diversity has been identified with sequencing methods than was previously known, due to the limitations of conventional identification methods that rely on shared metabolic characteristics (phenotypic tests) or gene sequencing, which typically only analyze a small fraction of the organism’s DNA. This has led to the taxonomic re-classification of entire genera (Yu et al., 2021). The increased use of next generation sequencing also enhances the speed and efficiency of bacterial identification methods, whereas conventional methods were more time-consuming and provided low resolution (Carleton and Gerner-Smidt, 2016; Besser et al., 2018; Gerner-Smidt et al., 2019a; Gerner-Smidt et al., 2019b; Stevens et al., 2022).

Historically, DNA–DNA hybridization (DDH) had been the gold standard for determining prokaryotic species for taxonomic classification (Rossello-Mora and Amann, 2001; Richter and Rossello-Mora, 2009). Rossello discussed the prokaryotic species concept in 2001, “Today, the accepted species classification can only be achieved by the recognition of genomic distances and limits between the closest classified (DNA–DNA similarity), and of those phenotypic traits that are exclusive and serve as diagnostic of the taxon (phenotypic property; Rossello-Mora and Amann, 2001).” This species concept is still applicable today; however, the genomic comparisons are now based on whole genome sequence (WGS) analysis.

In 2005, the average nucleotide identity (ANI) method was shown to be a plausible substitute for DDH since a 70% DDH threshold for species classification correlated well with a 94% ANI similarity threshold. This method, proposed by Kostantinidis et al., used pairwise alignment (BLAST) to identify the best hits of shared orthologous gene content between genomes being compared, obtaining the ANIb values (Konstantinidis and Tiedje, 2004; Goris et al., 2007; Richter and Rossello-Mora, 2009; Rodriguez-R, 2016). However, a drawback of ANIb is the need to perform gene prediction on the assembly before an ANI score can be determined.

Later methods eliminated the need for this prediction step by using local alignments of sequences of varying length and similarity. In 2007, Goris et al. expanded on the ANIb method by generating 1,020 bp fragments of the query genome and compared the ANI between the fragments and a reference genome using BLAST (Goris et al., 2007). In 2009, Richter et al. implemented an ultra-fast alignment tool, which compared the entire WGS contigs between genomes using the nucmer alignment program in MuMMer software, to calculate ANI values, referred to as ANIm (Kurtz et al., 2004). Kurtz et al. provided a dnadiff wrapper, which compares the resulting output files from the nucmer alignment program, to simplify and summarize ANIm output metrics for the differences between two genomes (Kurtz et al., 2004). Jain et al. further developed ANI methods by implementing FastANI, which is a method based on the minHash algorithm and read mapping. FastANI, similar to ANIb, aims to identify reciprocal or orthologous mappings and has an 80% identity cutoff (Ondov et al., 2016; Jain et al., 2018). FastANI has shown results that are comparable to the previous methods but has significantly improved the overall runtime to just seconds (Jain et al., 2018). GAMBIT was recently described as a kmer-based method comparable in accuracy and speed to FastANI (Lumpe et al., 2023). GAMBIT computes Jaccard distances based on a subset of the genome’s kmers and, similar to FastANI, uses raw sequencing reads (Lumpe et al., 2023).

Additional methods for species and subspecies identification have also been described. Ribosomal MLST was described by Jolley et al. (2012), but this method requires gene prediction, unlike ANIm and FastANI (Jolley et al., 2012). More recently, a new method for ribosomal MLST nucleotide identity (r-MLST-NI) has been developed for classifying *Klebsiella* and *Raoultella* species and may be useful for identifying other bacterial species (Bray et al., 2022). Public health laboratories in the United States, including our laboratory, have transitioned to WGS analysis from conventional methods for identification and surveillance of enteric pathogens. For this transition, a rapid and an easily standardized method of species identification using WGS was needed, which could be easily integrated into the PulseNet national molecular surveillance system [National Center for Emerging and Zoonotic Infectious Diseases (NCEZID), 2021] for enteric pathogens. In this study, we describe the implementation of an accurate, rapid, stand-alone, sequence-based method for the identification of *Campylobacter*, *Escherichia/Shigella*, *Listeria*, *Salmonella*, and *Vibrionaceae* species. This method is comparable to previous gold standard methods and utilizes the ANIm method. We compared over 450 genome assemblies to set the threshold ANIm values consistent with conventional identification methods. This method is currently employed for the precise speciation of enteric organisms from WGS using the Reference Genome Dataset version 2 (RGDv2) in BioNumerics and on the command-line, for routine identification of *Campylobacter*, *Escherichia/Shigella*, *Listeria*, *Salmonella*, and *Vibrionaceae* species.

## Materials and methods

### Genome selection for ANI detection

For this study, we selected two sets of genomes which included the Reference Genome Dataset version 2 (RGDv2, Supplementary Table 1) and the Test Genome Dataset version 1 (TGDv1, Supplementary Table 2). The strains were selected from genome assemblies available on NCBI or from the PulseNet Reference Outbreak Surveillance Team’s (PROST) enteric bacteria reference collections to represent the diversity of enteric bacteria. These well-characterized strains were previously identified by methods, such as phenotypic characterization, gene sequencing, phylogenetic analysis of the *rpoB* gene, and Accuprobe® (*Listeria monocytogenes*). All sequences met the standard PulseNet QAQC metrics, including a minimum Q score of 30, and sequencing coverages for downstream analysis: 40× for *Escherichia*, *Vibrio*, and *Shigella*, 30× for *Salmonella* and *Campylobacter*, and 20× for *Listeria* (Tolar et al., 2019).

The RGDv2 (Supplementary Table 1) included all species characterized as part of PulseNet, and the set was minimized for rapid analysis. It comprised 43 genome assemblies representing 32 enteric species, consisting of 10 assemblies representing 6 *Campylobacter* spp., 3 assemblies representing 3 *Escherichia/Shigella* spp., 11 assemblies representing 6 *Listeria* spp., 2 *Salmonella* assemblies representing 2 species, and 15 *Vibrionaceae* assemblies representing 11 *Vibrio* species, 1 *Grimontia* species, and 1 *Photobacterium* species. The RGDv2 assemblies were sequenced by Illumina, PacBio, or both instruments. The WGS reads for RGDv2 references were assembled using SPAdes for Illumina data (Bankevich et al., 2012) and HGAP (University M, 2014) for PacBio data. *Escherichia* and *Vibrio* genomes are larger and more complex due to phage regions; these assemblies were generated using both Illumina and PacBio sequencers. The NCBI BioSample data include additional information regarding sequencing chemistry and assembly methods for all strains.

The TGDv1 consists of 454 genome assemblies from 42 different enteric bacterial species (Supplementary Table 2), including the RGDv2 genome assemblies, and it is designed to represent all species necessary for querying identification, as well as rare and closely related species, to confirm the accuracy of ANIm for correct identification of species. The TGDv1 genomes were assembled using SPAdes v3.11 with default options (Bankevich et al., 2012).

### Development of custom ANI scripts

We developed custom scripts to utilize the dnadiff workflow in MUMmer v3.23 (Kurtz et al., 2004), facilitating pairwise comparisons with references and generating results in a tabular format. These scripts were developed for the command line. These scripts are published on our GitHub site (NCEZID-biome, 2021). The ANIm script runs on dnadiff and parses the field “AvgIdentity” to detect the percent identity. Additionally, to measure the breadth of the alignment, the script parses the AlignedBases field. To ensure consistency, the same ANIm script runs on both the command line (ani-m.pl) and as a plugin for BioNumerics (ani-m-bionumerics.pl).

### Determination of ANI metrics

The TGDv1 genomes were supplied as the reference and the query; the genomes were compared in a pairwise, all-vs-all fashion. The RGDv2 genomes, our gold standard set of references, were included in TGDv1 and the threshold analysis.

We used the ggplot2 and dplyr modules in R to analyze and generate a scatter plot of the values for ANI and percent aligned bases for all comparisons. Additionally, a violin plot was created from the ANI values for a subset of species represented in RGDv2. For the violin plot, only ANI comparisons with a minimum of 70% aligned bases were examined to ensure that percent ANI was being calculated over significant portions of the genome and to avoid spurious high percent ANI matches over repetitive regions.

### Down sampling for limits of detection

The reads for representative species of RGDv2 including two *Campylobacter*, three *Escherichia*, one *Listeria*, two *Salmonella*, and three *Vibrio* were downsampled to various coverage levels: 0.5×, 1×, 5×, 10×, 15×, 20×, 30×, 40×, and 50×. A 1× coverage was calculated as the total assembly size of the original coverage SPAdes assembly. The desired coverage and the total number of bases in the raw reads were used to calculate a percentage of the reads needed for that coverage level. Subsequently, we used the Fasten package (lskatz, 2023) to sample enough reads to meet the expected coverage. The coverage level was verified using the read metrics script in CG-Pipeline (Kislyuk et al., 2010). These downsampled reads were used to assemble each genome as previously described in this study. Most genomes at 0.5× and 1× could not be assembled with SPAdes and could not be used as assemblies for the 0.5× and 1× coverage level analyses.

At each downsampling level of every genome, we recorded the N50, a standard assembly metric. Then, we computed the ANIm method against the reference genome for each coverage level. We noted the change in the ANI value received at the different coverage levels as compared to the 50× downsampled assembly.

### Comparison of ANI methods: time trials and method compatibility

Pairwise ANI comparisons were generated using TGDv1 genomes, which were run in an all-vs-all fashion using the ANIm, FastANI, and ANIb algorithms, to evaluate the amount of time each method took from the launch of the script to report of the result. This workflow is encoded on our GitHub site (NCEZID-biome, 2021) as the *launch_all_ani* shell script. For each algorithm, we computed the ANI value and recorded the duration of each analysis using GNU time. Pairwise scatterplots for each pair of algorithms were plotted using ANI results, and a trend line was computed in Microsoft Excel; only algorithm pairs involving ANIm were included. Additionally, the frequency of the analysis durations for each algorithm were computed and plotted in Microsoft Excel.

## Results

### Determination of ANI metrics

**Figure 1:**
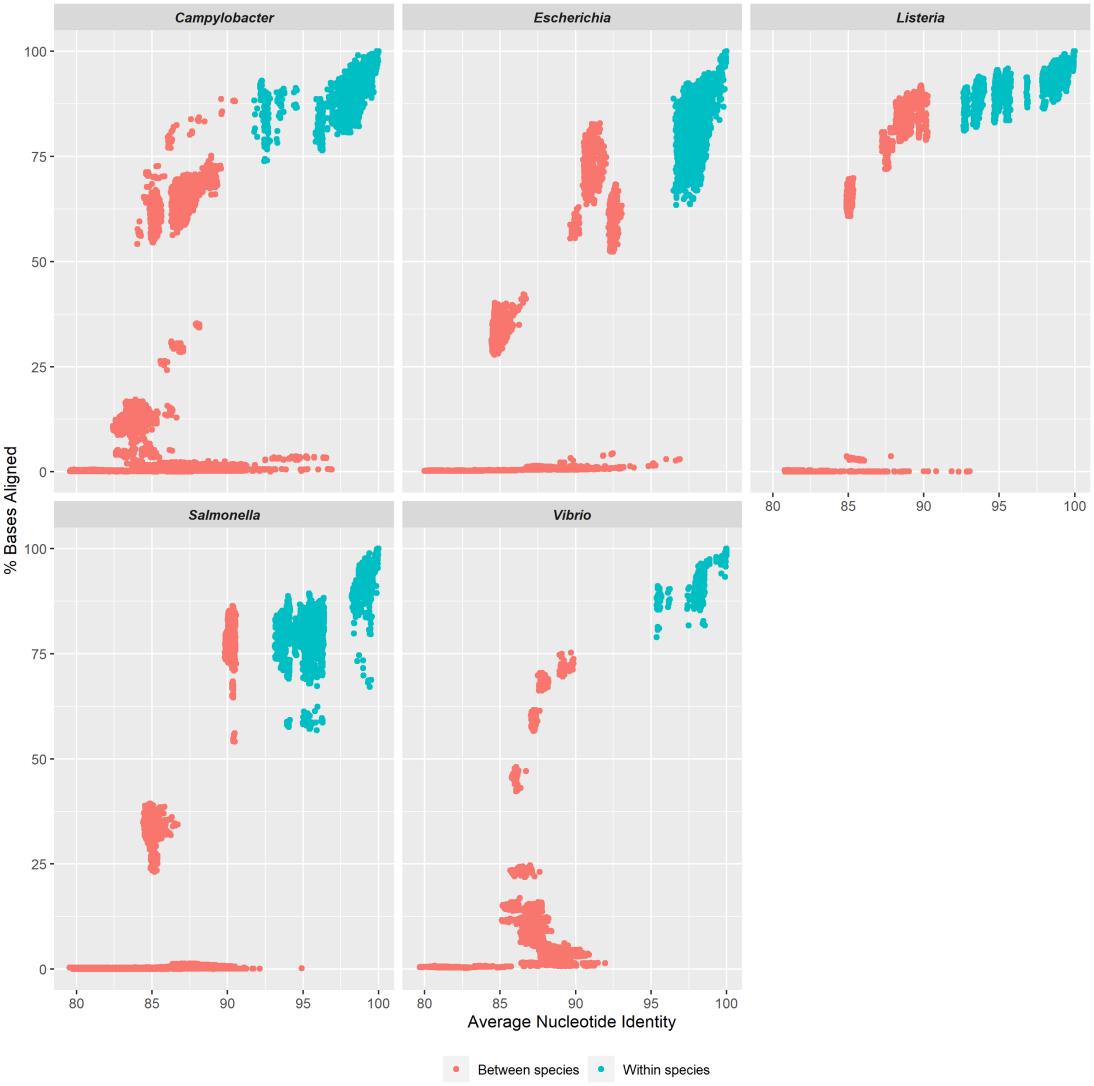
ANI limits for enteric detection. Scatter plots of average nucleotide identity versus percent aligned bases for four genera and one family: *Campylobacter*, *Escherichia*, *Listeria, Salmonella*, and *Vibrionaceae*. Each plot displays the relationship between ANI and percent aligned bases (e.g., reference genome alignment coverage) for both within-species and between-species in each group.

Computing the ANI of a query genome against a reference genome yields both the ANI value and the percentage of bases aligned. The percent bases aligned metric conveys what percentage of the reference genome is shared with the query. In this study, we compared the 454 TGDv1 genome assemblies in an all-vs-all comparison using ANIm (Supplementary Table 2), which resulted in 206,116 total comparisons. We plotted the percent bases aligned against the ANI for all genera and color-coded the between-species and within-species values (Figure 1). We noted that all the within-species ANI values appeared when the percent bases aligned was above 70%, consistent with our percent bases aligned threshold for excluding spurious high ANI matches.

**Figure 2:**
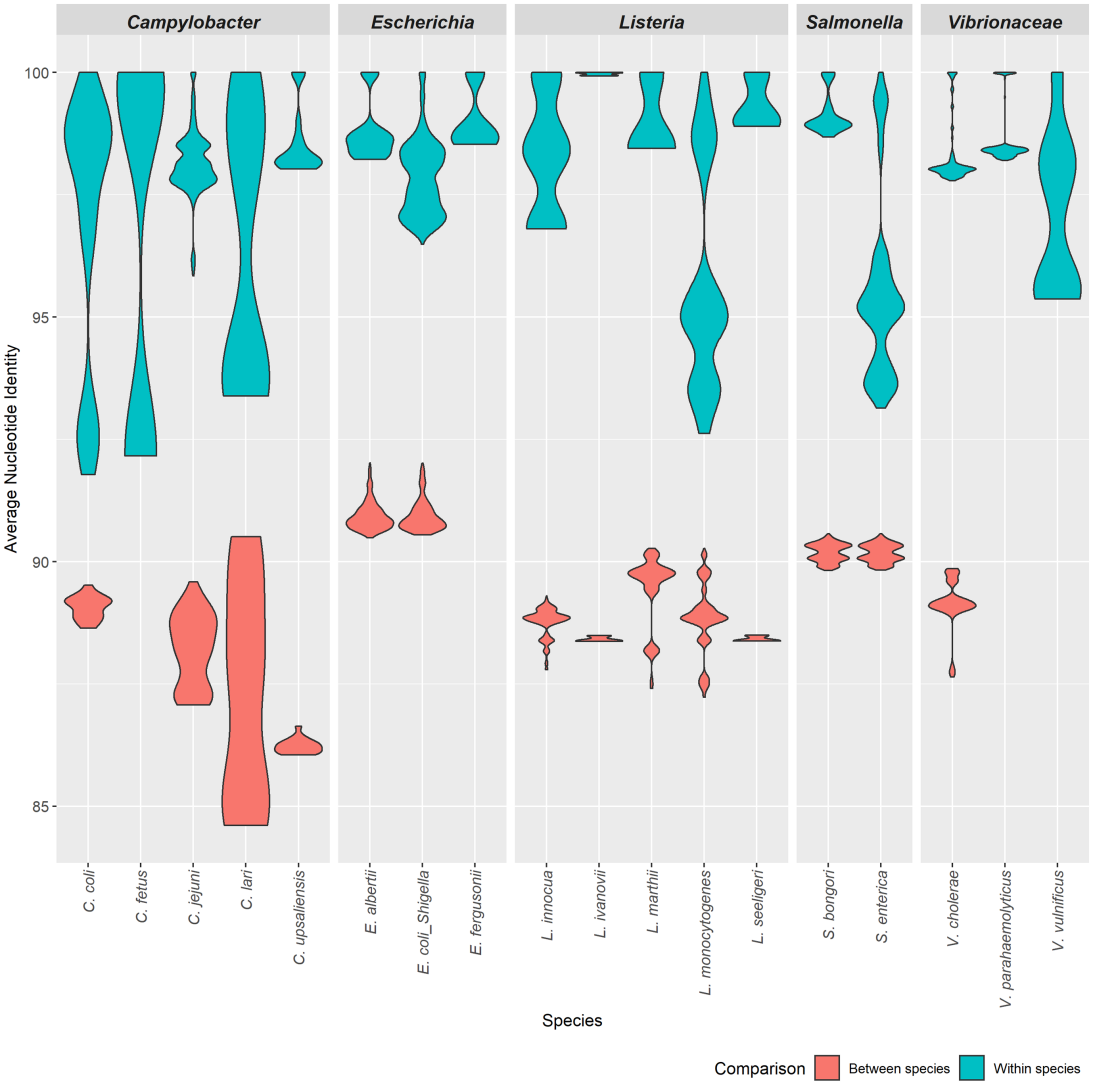
ANI values for five genera. Violin plots show ANI ranges for five genera: *Campylobacter*, *Escherichia*, *Listeria, Salmonella* and *Vibrio*. Each plot displays the variation in ANI values for both within a species (blue) and between a species (red) in each group.

By plotting all-vs-all ANI, we observed that the ANIm method effectively distinguished within-species comparisons from between-species comparisons, enabling the determination of thresholds for relevant species (Figure 2). The ANI threshold values were ≥ 95% for *Escherichia*/*Shigella* and *Vibrionaceae* species, ≥93% for *Salmonella* species, and ≥ 92% for *Campylobacter* and *Listeria* species; the ANIm method accurately classified all validation strains in the TGDv1 at the species level, when considering comparisons across >70% of bases aligned (Table 1). In this study, we identify an ANI threshold for each genus as shown in Table 1 based on the results of the ANIm analysis. Notably, *Vibrionaceae* and *Escherichia* species have a 95% threshold, while species from *Salmonella*, *Campylobacter*, and *Listeria* have a lower ANI threshold for distinguishing within-species from between-species comparisons (92–93%) when a ≥70% alignment threshold is met. We used traditional taxonomic definitions of these species that rely on phenotypic tests to verify these within-species and between-species comparisons (Ciufo et al., 2018). Some of these lower ANI thresholds may be the attributed to the greater diversity that WGS-based methods can capture compared to the conventional naming of *Salmonella*, *Campylobacter*, and *Listeria* species.

**Table 1:** Taxon-specific values for identification by ANI.

Taxon	ANI value (%)	Aligned bases (%)	Genome size (Mb.)
*Campylobacter* spp.	≥92	≥70	1.4 to 2.2
*Escherichia* spp.	≥95	≥70	4.5 to 5.5
*Listeria* spp.	≥92	≥70	2.7 to 3.1
*Salmonella* spp.^1^	≥93	≥70	4.56 to 5.5
*Vibrionaceae* spp.	≥95	≥70	3.8 to 6.2

Species level identification results are reported for query assemblies with ANI values listed below for *Campylobacter*, *Escherichia*, *Listeria, Salmonella*, and *Vibrionaceae* species. Taxon, ANI value (% value for ANI lower cutoff), aligned bases (%) and genome size (in megabases) for each species are listed. 1ANI can be used to identify one clinically important subspecies, *Salmonella enterica* subspecies enterica when the ANI score against the *Salmonella enterica* reference is >98%. Individual species thresholds may ultimately differ for *Salmonella bongori*, as all isolates tested to date result in >98% ANI score, >85% coverage, and lengths up to 5.0 Mb.

### Down sampling for limits of detection

**Figure 3:**
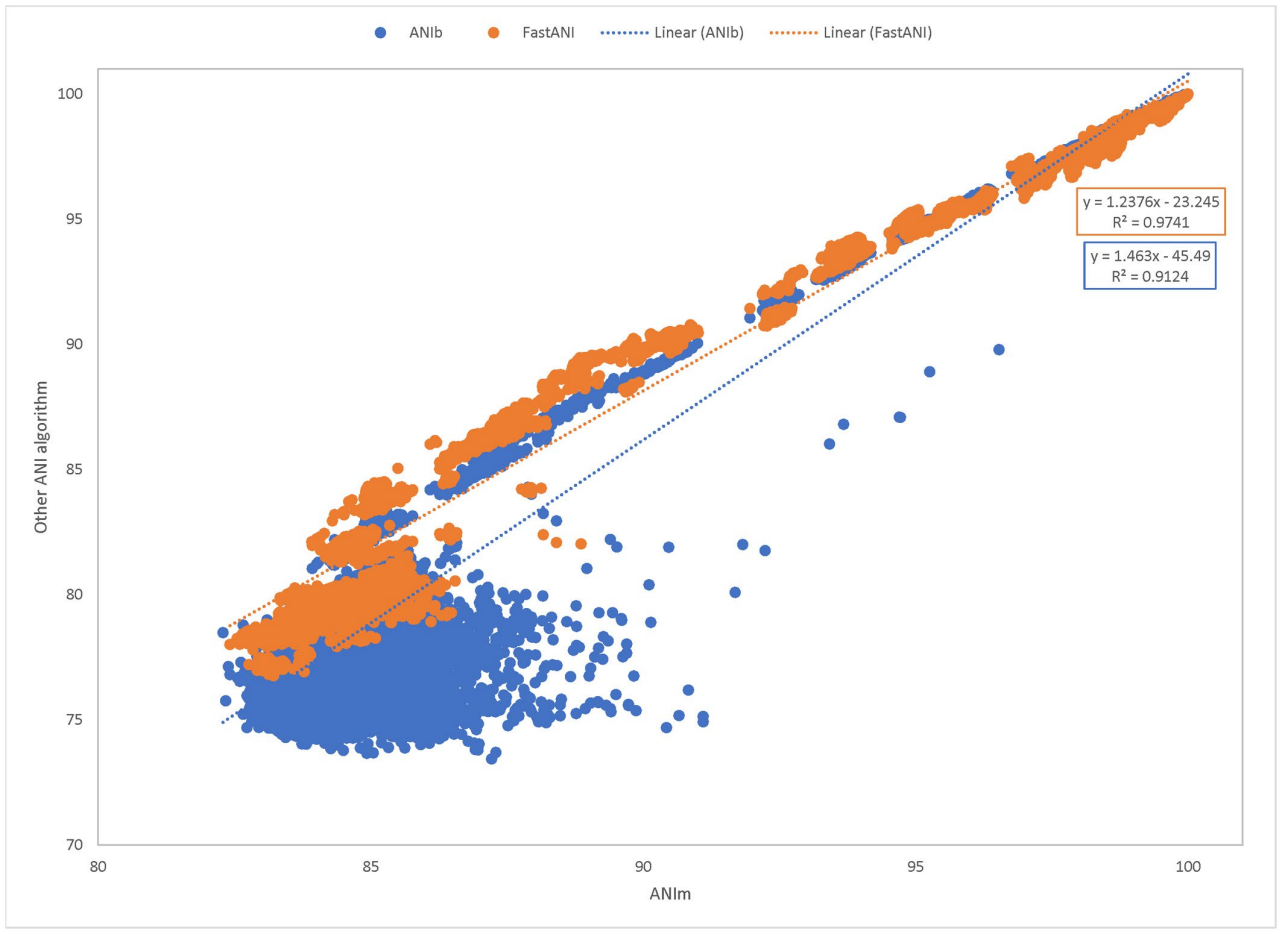
Downsampling for limits of detection. Representative species of *Campylobacter*, *Escherichia*, Listeria, *Salmonella*, and *Vibrio* were downsampled from 50× to 0.5× and analyzed with the ANIm algorithm. Genome coverage is plotted on the x-axis; the natural log of N50 (lnN50) is plotted on the left y-axis; and percent change from ANI at 50× is plotted on the right y-axis. The dotted blue line shows the average N50 for all the assemblies. The dark green line indicates the aggregate ANI values, or the average percentage that each ANI value deviated from what it was at 50×. Coverage cutoff of 10× was established based on this analysis, as species identification is not reliable below 10×. Additionally, the aggregate ANI begins accruing below 10×, gaining larger standard deviations.

To determine the robustness of the ANIm method at different coverage levels, an experiment was conducted to determine the lowest depth of coverage of a genome assembly required for accurate species identification. Several assemblies from representative species were assembled from coverage depths of 50× to 0.5× to find where an ANI value starts deviating (Figure 3). After down sampling, most genomes at 0.5× and 1× could not be assembled with SPAdes. In some cases, identification was made at 5× coverage, especially for *Salmonella* and *Listeria* genomes. For all enteric species in RGDv2, we determined a minimum of 10× depth-of-coverage for genome assemblies. In the standard bioinformatic analysis for molecular surveillance within PulseNet, the sequencing depth cutoffs are 40× for *Escherichia*, *Vibrionaceae* and *Shigella*, 30× for *Salmonella* and *Campylobacter*, and 20× for *Listeria*, which makes ANIm compatible with this public health usage (Tolar et al., 2019).

### Comparison of ANI methods: time trials and method compatibility

**Figure 4:**
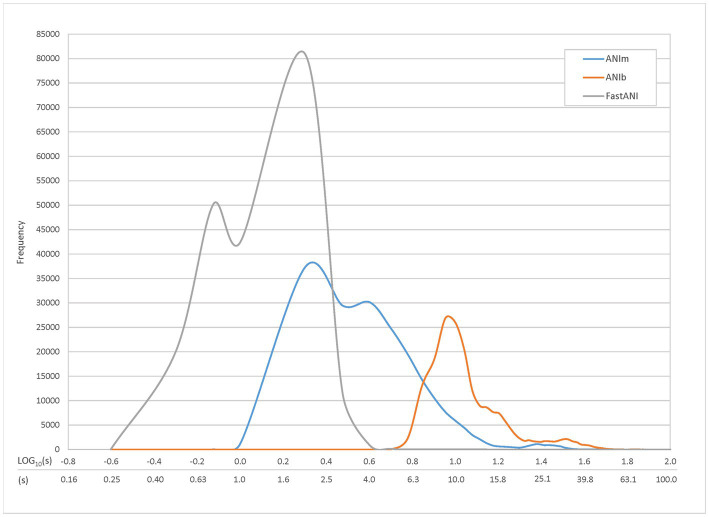
Individual Query Speed by ANI Method. Time trials were conducted to compare the runtime of three different ANI methods: ANIb, ANIm, and FastANI. TGDv1 genomes were compared against each other, and 206,116 total comparisons were generated along with their associated runtimes. Approximately 0.10% (ANIm) and 0.02% (ANIb) of the comparisons were excluded because they exceeded the maximum graphical runtime of 100 s; there were no comparisons excluded for FastANI. The most common runtimes were approximately 9 s for ANIb, 2 and 4 s for ANIm, and 0.75 and 2 s for FastANI; two different frequency peaks were noted for ANIm and FastANI.

We compared several methods to calculate ANI: ANIb, ANIm, and FastANI. We first compared these three methods in a speed trial (Figure 4), examining the range of ANI runtimes for pairwise comparisons. An all-*vs*-all comparison of the TGDv1 showed that FastANI trials produced the fastest results, followed by ANIm and ANIb. Peak frequency runtimes for FastANI (approximately 0.75 and 2 s), ANIm (approximately 2 and 4 s), and ANIb (approximately 9 s) were observed; two different frequency peaks were noted for ANIm and FastANI. FastANI, while being an order of magnitude faster than ANIm, lacks an alignment report that includes the number or percentage of aligned bases, similar to ANIb. We selected ANIm as a preferred method due to speed, and it has provided the desired output of ANI score and percent genome alignment.

**Figure 5:**
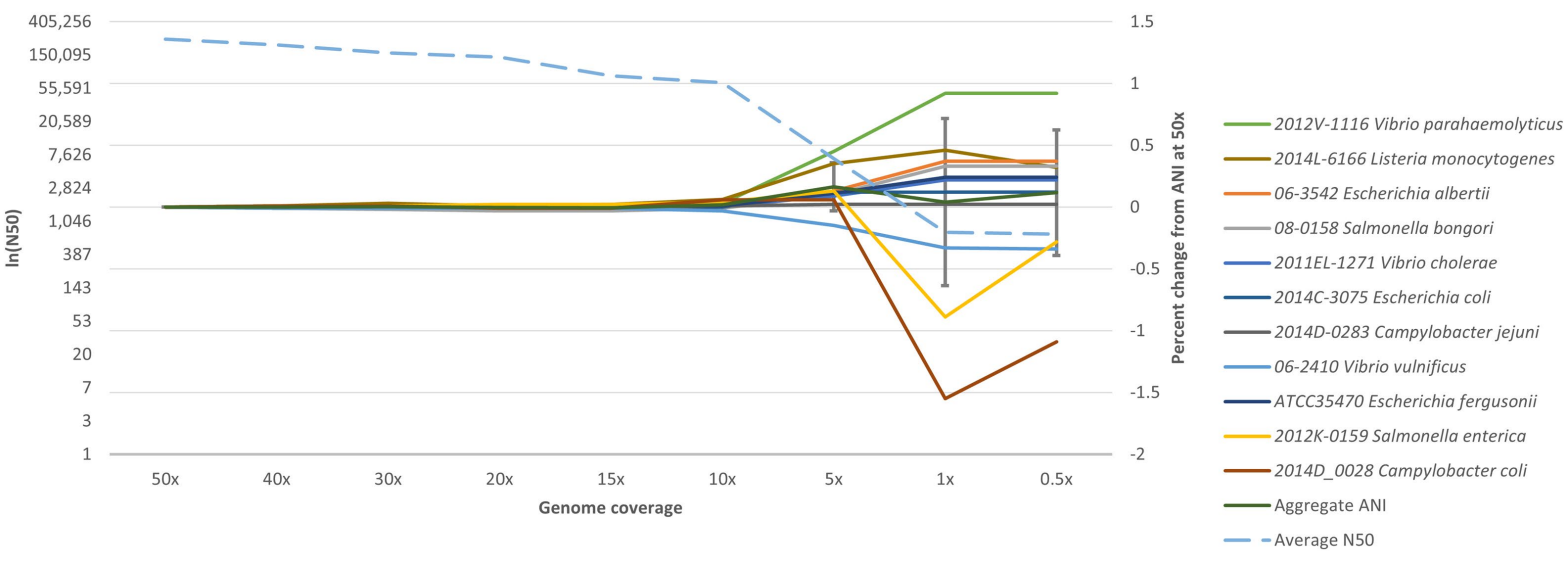
Pairwise comparisons of ANIb and FastANI to ANIm. ANIm is plotted on the x-axis while ANIb and FastANI are plotted on the y-axis. All data satisfied the ANIm metric of greater than 70% aligned bases. A goodness-of-fit was detected for each method. FastANI’s slope is close to one (FastANI: y = 1.2376–23.245with an R^2^ = 0.9741), while ANIb’s slope is also close to one (ANIb: y = 1.463× − 45.49 with an R^2^ = 0.9124).

Using the same results from the time trials, we next measured the similarity between the results when comparing FastANI to ANIm and ANIb to ANIm (Figure 5). We plotted the percent identity of ANIb and FastANI against ANIm to form a scatterplot. This benchmark shows a trendline with FastANI: y = 1.2376× − 23.245 (R^2^ = 0.9741) and ANIb: y = 1.463× − 45.49 (R^2^ = 0.9124). The R^2^ scores suggest a correlation between ANIb, ANIm, and FastANI. However, ANIb and FastANI often reported ANI scores of 0, a null value, when compared against distantly related species; instances of null ANI scores were excluded in our benchmark analysis. ANIb and FastANI do not consider low identity regions in their calculations, and ANIb and FastANI report these null ANI scores when the scores fall below 60 and 80%, respectively (Konstantinidis and Tiedje, 2005; Jain et al., 2018). Alternatively, ANIm does not have this requirement and null ANI values were never reported for ANIm.

When removing null percentages, ANIb scores ranged from 73.43 to 100.00 with Q1, median, and Q3 being 77.01, 79.55, and 89.00, respectively (Supplemental Table 3). Similarly, FastANI scores ranged from 76.76 to 100.00 with a median of 82.15, Q1 of 81.75, and Q3 of 95.11. Similarly, the associated ANIm scores ranged from 82.42 to 100.00 with a median of 84.98 (Q1 and Q3: 84.47 and 95.23) for the FastANI trendline and ANIm scores from 82.29 to 100.00 with a median of 85.15 (Q1 and Q3: 84.45 and 90.21) for the ANIb trendline. Inclusion of additional ANIm scores, which were associated with null percentages in either ANIb or FastANI, had an adjusted range of 78.51100.00 with a median of 83.48, Q1 of 81.53, and Q3 of 85.6 (Supplemental Table 3).

**Figure 6:**
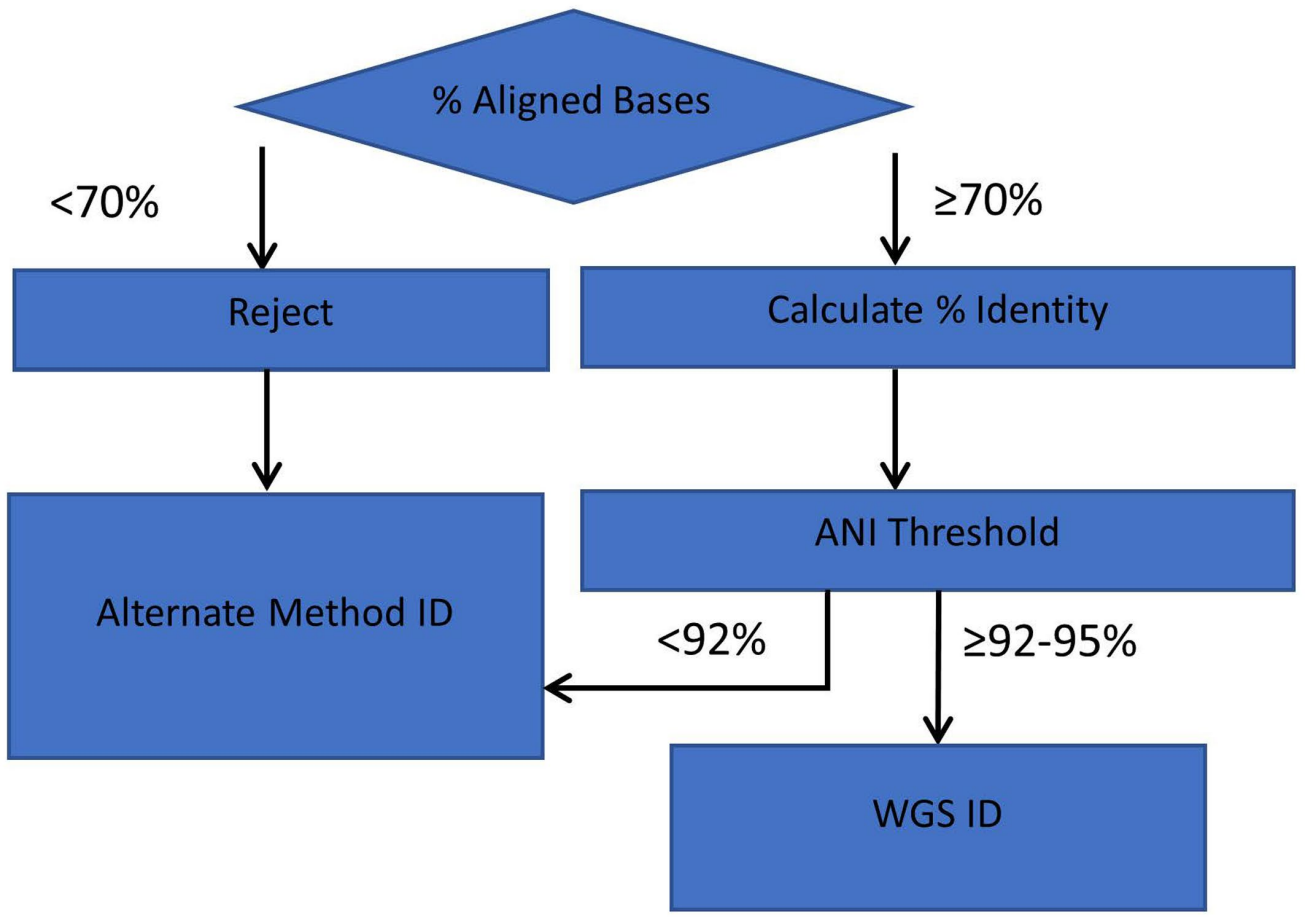
Workflow diagram for ANI method of identification. Genome comparisons with >70% of aligned bases and lower ANI Thresholds (≥92–95%, depending on species) are acceptable for interpretation and reporting with the ANI identification workflow.

An outline of the ANIm species identification method is illustrated in Figure 6. For routine identification, ANI values are calculated for genome assemblies that meet or exceed the alignment criteria of 70% aligned bases with an RGDv2 reference(s). If the threshold meets the cutoffs per species (Table 1), then a species identification is reported.

## Discussion

The ANIm method described here allows for rapid, quantitative, and accurate species identification using the WGS data from enteric bacteria. We have implemented an ANIm methodology on the UNIX command line and in BioNumerics version 7.6 for routine identification of *Campylobacter*, *Escherichia/Shigella*, *Listeria*, *Salmonella*, and *Vibrionaceae* species. The ANIm value and percent bases aligned describe the extent to which one genome assembly is identical to another and can be used to determine the species identity of an assembled query genome by comparing it to a database of reference genomes with historically described taxonomy. To generate this reference genome database for ANIm species identification, we assembled the RGDv2, which contains 43 high-quality representative genomes for relevant PulseNet species, whose species identity had been established with previous gold standard methods (Supplementary Table 1). Any genome assembly can be compared against the reference genomes found in the RGDv2 for species identification. This smaller representative set of reference genomes was chosen to make this identification faster. To expand ANI speciation to other species, a representative genome or genomes of the species of interest, after validation, can be added to the RGDv2 (Supplementary Table 1).

We determined the thresholds for species identification with ANIm by comparing the enteric bacterial genomes from TGDv1, which comprised 454 genomes, including the RGDv2 genomes, whose species identity had also been previously established using gold standard methods. The analysis showed that ANI threshold values of ≥95% for *Escherichia*/*Shigella* and *Vibrionaceae* species, ≥93% for *Salmonella* species, and ≥ 92% for *Campylobacter* and *Listeria* species classified all validation strains in TGDv1 accurately at the species level, when considering comparisons across >70% of bases aligned. The ANIm thresholds reported in this study are similar to the previously published species boundaries for ANIb (94%), ANIm (95–96%), and FastANI (95%; Konstantinidis and Tiedje, 2005; Richter and Rossello-Mora, 2009; Jain et al., 2018). The lower ANI boundaries (92–93%) observed in this study for *Salmonella*, *Campylobacter*, and *Listeria* may be due to a wider degree of diversity within the species of those genera. As new species may be identified for these genera, we will re-evaluate our ANI thresholds. Moreover, we performed downsampling experiments to examine how genome coverage levels affect the ability of the ANIm tool to provide a result consistent with gold standard methods, and we found that reliable speciation using ANIm can be achieved with genomes assembled from ≥ sequencing read coverage of 10× or greater.

We compared three different methods for computing ANI: ANI using BLAST (ANIb), ANI using MuMMer (ANIm), and FastANI. We focused our comparison on these ANI methods and evaluated them for speed, accuracy, and easy interpretation. While all three of the ANI methods tested were comparable in speed and accuracy, ANIm was the easiest to standardize and interpret using the ANI and percent bases aligned metrics provided by the dnadiff wrapper script. We compared ANIm to ANIb and FastANI by correlating the ANI values from pairwise comparisons across the TGDv1 genome set. All three methods produced comparable ANI results with correlation coefficients of 1.24 and 1.46 and high R^2^ scores (>0.9), for both the correlation of FastANI to ANIm and ANIb to ANIm. Additionally, we evaluated the differences in speed of the three distinct tools. All three of the ANI methods had median run times of less than 10 s for a pairwise comparison. To the best of our knowledge, this is the first comparison of the runtime for ANIm and FastANI. FastANI analyses were generally completed faster than ANIm and ANIb, and ANIm was somewhere in the middle from job submission to result. However, overlap was observed in runtimes among all three tools. As all tools demonstrate efficient performance within the range of 10 s or less, the variations in runtimes are likely not significant until a large number of comparisons are being analyzed. While other methods, such as ribosomal MLST, ribosomal MLST nucleotide identity (r-MLST-NI), and k-mer based methods like GAMBIT, hold promise for bacterial species identification, it is important that these methods were not evaluated in this study.

In this study, we have implemented ANI for enteric species identification using MUMmer (ANIm) and demonstrated the utility of ANI for species identification. Furthermore, we simplified ANI-based enteric species identification using a new standard database, RGDv2, built from reference genomes identified with previous gold standard methods and demonstrated its robustness. We also showed that only 10**×** sequencing coverage is needed to reliably detect species using RDGv2. This low coverage requirement and the speed of the ANIm analysis are advantageous when turnaround time is crucial, as is common in public health settings. For further variant analysis, we have higher coverage requirements in PulseNet. An opportunity for future development may include evaluating the robustness of ANI with additional genome assembly methods compatible with both short-and long-read sequencing methods. The approach here is also generalizable for any situation, where a set of organisms need to be rapidly identified for species by adding and validating reference species genomes to an ANIm database.

## Data availability statement

The datasets presented in this study can be found in online repositories. The names of the repository/repositories and accession number(s) can be found in the article/Supplementary material.

## Author contributions

LG, TG, AH, LK, and RL: conceptualization, validation, visualization, and writing—review and editing. LG, AH, and LK: software. TG: data curation. LG, AH, LK, and TG: formal analysis. CL, PS, MI, BD, and ZK: investigation. RL: project administration and writing original draft. HC: final review.
