# Supplementary material for: Rapid identification of enteric bacteria from whole genome sequences using average nucleotide identity metrics
Source: Front Microbiol. 2023 Dec 14;14:1225207. doi: 10.3389/fmicb.2023.1225207 (PMC10752928; doi:10.3389/fmicb.2023.1225207)
Supplement: Supplementary file 3 [file Data_Sheet.PDF]

Supplementary Material

Rapid identification of Enteric Bacteria from Whole Genome Sequences using Average Nucleotide Identity

Rebecca L. Lindsey*, Lori Gladney, Andrew Huang, Taylor Griswold, Lee S. Katz, *et.al*

*** Correspondence:** Rebecca Lindsey: rlindsey1@cdc.gov

1 Supplementary Table 1. Reference Genome Dataset version 2 (RGDv2)

RGDv2 consists of 43 Reference Genomes used for ANI speciation. The species, subspecies, NCBI BioSample, NCBI assembly and strain identification number are listed for all genomes. Attached excel file.

2 Supplementary Table 2 (TGDv1)

A list of the 454 genome assemblies in the Test Genome Dataset (TGDv1). Information includes strain name, organism, the NCBI BioSample Accession number, and GenBank Accession for the assemblies used in this study. Attached excel file.

3 Supplemental Table 3 (ANI Method Statistics)

Common statistical values for the different ANI methods (ANIb, ANIm, and FastANI) generated from the Figure 5 ANI scores dataset. Inclusion (= 0) or exclusion (≠ 0) of null or null-associated ANI scores has been notated where appropriate. Types of statistical values include minimum, first quartile, median, third quartile, and maximum.

ANI Method Statistics					
	ANIm **	ANIb/ANIm (Figure 5)		FastANI/ANIm (Figure 5)	
		ANIm	ANIb (≠ 0)	ANIm	FastANI (≠ 0)
Min	78.51	82.29	73.43	82.42	76.76
Q1	81.53	84.45	77.01	84.47	81.75
Median	83.48	85.15	79.55	84.98	82.15
Q3	85.6	90.21	89.00	95.23	95.11
Max	100	100	100	100	100.00

**: Includes all available ANIm scores including any associated with ANIb (= 0) and/or FastANI (= 0).
